# Lymphocytes Change Their Phenotype and Function in Systemic Lupus Erythematosus and Lupus Nephritis

**DOI:** 10.3390/ijms252010905

**Published:** 2024-10-10

**Authors:** Eleni Moysidou, Michalis Christodoulou, Georgios Lioulios, Stamatia Stai, Theodoros Karamitsos, Theodoros Dimitroulas, Asimina Fylaktou, Maria Stangou

**Affiliations:** 1 School of Medicine, Aristotle University of Thessaloniki, 54124 Thessaloniki, Greece; moysidoueleni@yahoo.com (E.M.); michalischristodoulou22@gmail.com (M.C.); pter43@yahoo.gr (G.L.); staimatina@yahoo.gr (S.S.); tkaramitsos@auth.gr (T.K.); dimitroul@auth.gr (T.D.); 2 1st Department of Nephrology, Hippokration General Hospital, 54642 Thessaloniki, Greece; 3 1st Department of Cardiology, AHEPA University Hospital, 54636 Thessaloniki, Greece; 4 4th Department of Medicine, Hippokration General Hospital, 54642 Thessaloniki, Greece; 5 Department of Immunology, National Histocompatibility Center, Hippokration General Hospital, 54642 Thessaloniki, Greece; fylaktoumina@gmail.com

**Keywords:** systemic lupus erythematosus, adaptive immunity, B and T lymphocytes, phenotype, function

## Abstract

Systemic lupus erythematosus (SLE) is a complex autoimmune disease, characterized by considerable changes in peripheral lymphocyte structure and function, that plays a critical role in commencing and reviving the inflammatory and immune signaling pathways. In healthy individuals, B lymphocytes have a major role in guiding and directing defense mechanisms against pathogens. Certain changes in B lymphocyte phenotype, including alterations in surface and endosomal receptors, occur in the presence of SLE and lead to dysregulation of peripheral B lymphocyte subpopulations. Functional changes are characterized by loss of self-tolerance, intra- and extrafollicular activation, and increased cytokine and autoantibody production. T lymphocytes seem to have a supporting, rather than a leading, role in the disease pathogenesis. Substantial aberrations in peripheral T lymphocyte subsets are evident, and include a reduction of cytotoxic, regulatory, and advanced differentiated subtypes, together with an increase of activated and autoreactive forms and abnormalities in follicular T cells. Up-regulated subpopulations, such as central and effector memory T cells, produce pre-inflammatory cytokines, activate B lymphocytes, and stimulate cell signaling pathways. This review explores the pivotal roles of B and T lymphocytes in the pathogenesis of SLE and Lupus Nephritis, emphasizing the multifaceted mechanisms and interactions and their phenotypic and functional dysregulations.

## 1. Introduction

Systematic lupus erythematosus (SLE) is generally described as a prototypic chronic autoimmune disease characterized by a disturbed interplay between innate and adaptive immune system with loss of self-tolerance and auto-antibody production [1].

It mainly affects female patients (10 women for every man) of reproductive age, while incidence and prevalence vary and depend on factors like age, gender, ethnicity, classification criteria, etc. [2]. SLE is clinically characterized by heterogeneity; it can have a wide range of effects on any organ, frequently with non-specific manifestations like arthritis and fever or uncommon and rare effects like autoimmune haemolytic anaemia, neurological manifestations, antiphospholipid syndrome, etc. In certain cases, SLE might be diagnosed based on a single organ injury [2,3,4]. As a result, so far, we lack adequate diagnostic criteria, and diagnosis is based on a number of clinical and laboratory findings. Following proper immunosuppressive therapy, the disease will eventually go into remission, with a long course of flare-ups and remissions, adding to organ damage [2].

Lupus nephritis is a type of glomerulonephritis and one of the severest manifestations of SLE [5]. It may affect up to half of all patients during diagnosis of SLE or later. Indications of possible kidney involvement are hematuria, proteinuria, and nephritic (hematuria, proteinuria, high blood pressure, edema) or nephrotic syndrome (proteinuria, hypoalbuminemia, edema). Kidney biopsy is the gold standard method for diagnosis and classification, providing to the nephrologist important data regarding prognosis and required treatment [6]. Eventually, about 20% of affected patients will develop end stage renal disease [7].

In certain individuals, the combination of genetic, epigenetic, and environmental parameters contribute to the progression of autoimmunity and production of antinuclear antibodies (ANA), already present at very early stages of the disease, not necessarily leading to a clinical syndrome [3,8]. This indicates that other mechanisms, apart from autoantibodies and immune-complex (IC) formation, are involved in the pathogenesis and clinical presentation of SLE. Activation of the innate immune system is always present, as demonstrated by the release of neutrophil extracellular traps (NETs), impaired clearance of ICs and apoptotic cells, activation of complement, type I interferons (IFNs), and interleukins (ILs) pathways, and alteration in Toll-like receptors (TLR) activity [3,9,10].

In particular, the process of cell death is a common natural mechanism affected by SLE. Impaired clearance of apoptotic products and debris in terms of necrosis lead to release of endogenous antigens and danger associated molecular patterns (DAMPS) [11]. These nucleic acid containing products are recognized by pattern recognition receptors (PRRs) and more specifically TLRs on plasmacytoid dendritic cells (pDCs), mainly type 7 and 9 that are in fact overexpressed in SLE [12], and finally end up activating endocytic signal pathways. As a result, different types of inflammatory cells are recruited (monocytes, macrophages, neutrophils, dendritic cells) as well as chemokines and cytokines such as type I IFN [13]. Furthermore, in SLE patients, some of the characteristic disorders of neutrophils are aberrant phagocytosis, increased apoptosis, and reduction of their number in circulation and damage to their metabolism [14,15,16]. Τranscriptomic analysis in peripheral blood of SLE patients revealed, among different blood signatures, enrichment of neutrophil transcripts, which was related to disease activity and renal involvement [17]. In addition, the “IFN signature” of certain inflammatory subsets, the low-density granulocytes (LDGs), is brought to light from epigenomics [18]. All these data point the significant contribution of neutrophil in SLE autoimmunity.

Release of neutrophil extracellular traps (NETs), namely NETosis, is another mechanism of extracellular chromatin exposure [9]. This relatively new form of cellular death, discovered circa 20 years ago [19], and NETs were described to be abundant but also deficiently degraded in SLE patients due to the presence of antibodies against them and inhibitors of DNAase-1 [20]. Afterwards, these bearing self-antigens NETs, in a TLR-dependent manner, induce the production of type I IFN by pDC, the cells that present these self-antigens to T and B lymphocytes. Meanwhile accumulating data support their devastating effect on vascular damage, thrombosis, fibrosis, and their pathogenetic role in kidney involvement [9,20,21]. NETs themselves, and the autoantibodies against them, induce C1q deposition, leading to further DNase-1 inhibition, and enhancement of impaired degradation and inflammation [22]. C1q is classified among the factors, together with C2 and C4, that after mutation are able to cause monogenic forms of SLE [23]. Except from complement factors, other humoral elements of the innate immune system associated with SLE pathogenesis are ILs and type I IFN signaling [24,25]. A high “IFN-α signature” is found in most of the patients with SLE [26], whereas SLE development in animal models is improved after genetic ablation of type I IFN signaling [27]. Accumulation of immune-complexes provokes type I IFN production, mainly IFN-α, with pleiotropic effects inducing the disease, such as activating DCs, neutrophils, natural killer (NK) cells, and T helper (Th) lymphocytes, stimulating the production of auto-antibodies by B lymphocytes, improving the antigen presentation of DCs, and inducing the production of plenty of cytokines such as IL-6 [28]. All the above-mentioned factors, as well as activation of the complement system, cytokines including IFN type I, IFNγ, IL6, IL12, IL21, IL23, and cells of myeloid origin (neutrophils, monocytes etc.) additionally mediate local renal damage, promoting inflammation as well as endothelial dysfunction [3].

However, at the same time, the adaptive immune system is profoundly involved, as manifested by B cell dysregulation and its contribution to autoreactivity in terms of auto-antibody and cytokines production [29], and, in a more sophisticated way, through T lymphocyte immunity, as T lymphocytes interact erroneously with aberrant B lymphocytes, while their immunoregulation efficacy is definitely reduced [30]. Defects in all these mediators of the cellular and humoral response are proposed by genome-wide association studies (GWAS), in vitro studies, and murine lupus models, all designed to clarify a tiny portion of the complicated network of SLE pathogenesis [3,31]. In this review, we will highlight the hallmarks of B and T lymphocyte related dysregulations, which are pertinent to SLE and lupus nephritis pathogenesis.

## 2. Β Lymphocytes Related Abnormalities

The involvement of B cells in the pathophysiology of SLE was established as early as the late 1960s, when it was initially shown that serum from lupus patients contained self-reactive antibodies against nucleic antigens [32]. Since then, extended research revealed multiple ways that B lymphocytes contributed to the pathophysiology of SLE, including not only polyclonal B-cell hyper-reactivity and auto-antibody formation, but also antigen presentation and cytokine production [33,34,35,36].

### 2.1. Dysregulated Function of B Lymphocytes

#### 2.1.1. Loss of Self-Tolerance

Constitutional disorder in SLE is the loss of self-tolerance, which leads to the generation of auto-reactive B cells [37]. In the normal process of B cell evolution, a certain amount of naïve B cells in the peripheral blood display auto-reactivity instead of tolerance mechanisms such as deletion, receptor editing, or anergy [29,38]. This “silent” and inactive subset is falsely activated in SLE patients [39] through stimulation of certain types of receptors, including B-cell receptors (BCRs), TLRs, B-cell activating factor receptors (BAFF-Rs), and, accordingly, their related intracellular pathways [40].

Regarding BCRs, next generation sequencing (NGS) studies in case-control series featured upregulation of the transcription of VAV2 and phospholipase C-γ2 (PLC-γ2) (factors related to the BCR pathway and managing calcium entrance) [41,42] while the transcription of six other genes was downregulated (VAV1, VAV3, ΝFAT, AKT1, AKT3, IGH, GSK3β) [40,43,44,45,46,47,48,49,50].

An additional activating hit for the B cells are the TLRs, which are not only related to innate but also to adaptive immunity [51]. Endosomal TLR7 and 9, types of TLR that are located into cell endosomes and are meant for viral nucleic-acid identification [52], have been closely associated with SLE pathogenetic mechanisms [53,54,55]. In fact, numerous animal studies in which TLR7, TLR9, or the related signaling factor Myd88 was deleted demonstrated remission of related auto-antibody production in murine lupus, whereas transgenic over-expression provoked B cell stimulation and formation of germinal centers (GCs) [12,56,57,58,59]. Polymorphisms of genes correlated with both BCR and TLR signaling pathways have intensified their place into the SLE pathogenetic process. More specifically, GWAS defined BCR signaling genes PTPN22, BANK1, BLK, LYN, GRB2, PXK, and TLR signaling variants TLR7, TNFAIP3, TNIP1, UBE2L3, IRAK1,SLC15A4, and CXORF21 as being of high importance [60,61,62,63,64].

Apart from and in close connection with the previously mentioned mechanisms, B cell activating factor, or BAFF, is another crucial element for B cell survival and stimulation [65,66]. BAFF, also mentioned as B lymphocyte stimulator (BLys), and A proliferative-inducing ligand (APRIL) are members of the tumor necrosis factor (TNF) ligand family produced by cells of the myeloid lineage (DCs, macrophages, neutrophils) and critically interfere with B cell survival and differentiation [1,29]. These cytokines exert B cell survival, proliferation, and differentiation via binding to three certain B cell receptors: BAFF-R, a transmembrane activator, calcium modulator and cyclophilin ligand interactor (TACI), and B cell maturation antigen (BCMA) [67]. BAFF receptor expression promotes survival of B cells and has initially been detected in T1 transitional B cells [68].

Animal experiments exhibited lupus-like disease with increased circulating and kidney-depositioned ICs in BAFF transgenic mice where BAFF was over-expressed [69,70,71]. Deleting TACI receptor in these mice had a positive impact on aberrant antibody production and also protected them from progressive lupus glomerulonephritis [72]. Increased levels of BAFF were also found in SLE patients and studies have proven a close correlation with anti-dsDNA levels and disease activity [73,74]. BAFF dysregulates peripheral tolerance, promoting clonal proliferation of autoreactive B cell in a T cell independent way [65].

On the other hand, activity of B cells is controlled by inhibitory signals that also suffer in autoimmune diseases. Particularly, polymorphisms in genes of the human FcγRIIB, an inhibitory receptor on B cells, are associated with lupus phenotype [75,76]. Hence, it becomes clear that the break is loose and the throttle is pushed in SLE.

**Figure 1:**
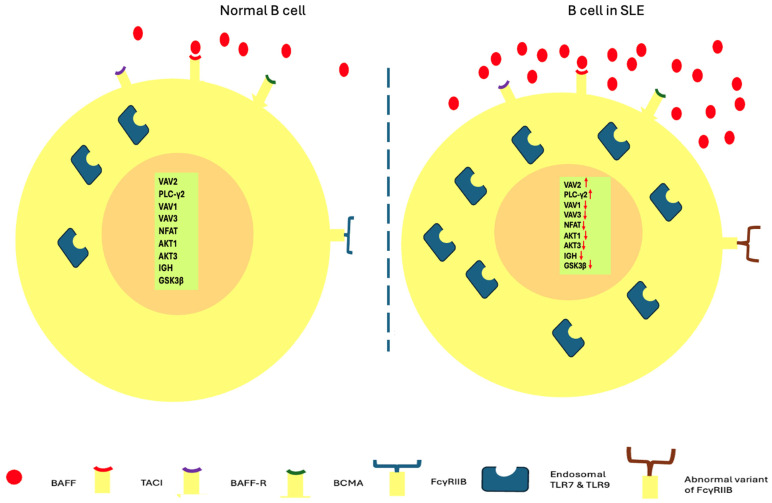
B cells in healthy status and SLE regarding loss of tolerance. Overexpression of BAFF, overactivation of endosomal TLR7 and 9, variants of FcγRIIB receptors, as well as dysregulated transcription of VAV2, PLC-γ2, VAV1, VAV3, ΝFAT, AKT1, AKT3, IGH, GSK3β are implicated in the abnormal tolerance of B lymphocytes (arrows pointing up: upregulation; arrows pointing down: downregulation).

The above B lymphocytes in SLE regarding loss of tolerance are depicted in Figure 1.

#### 2.1.2. Intrafollicular and Extrafollicular B Lymphocyte Activation

It is well described that autoantibodies present in SLE, especially anti-DNA antibodies, are produced through procedures that include somatic hypermutation and affinity maturation [77]. These traits suggest the domination of GC origination theory, in which T follicular helper cells and activated B cells interact within the GC to produce high-affinity, long-lived plasma cells that secrete antibodies [78]. However, in the effort to understand the pathogenetic pathways leading to antibody generation, research from animal models has revealed possible involvement of extrafollicular (EF) pathways, in both a T cell-dependent and T cell-independent response, outside the follicle [65,79]. Additionally, NGS analysis in human SLE patients displayed a fraction of effector B cells, the so-called activated naïve B cells (acN), that did not reach affinity maturation [80]. Consequently, antibody-secreting cells (ASC) derive from both pathways.

#### 2.1.3. Cytokine Production

There are several other mechanisms contributing to the apparent perturbation of B lymphocyte regulation in SLE. Besides the above-mentioned alterations that form hyper-reactive B cells, a wide range of cytokines are implicated in the pathogenesis of the disease. Higher levels of IL-6, a pro-inflammatory cytokine that stimulates B cell differentiation in an autocrine and paracrine way, were found in SLE patients. Downregulating antibody secretion was linked to blocking IL-6’s effect by blocking its receptor [81]. Additional involvement in the pathogenesis and progression of lupus nephritis has also been demonstrated in animal models [82,83]. This upregulation of IL-6 production is also provoked by IFN-γ signaling [84], and studies have positioned these events around the onset of autoantibody production, elucidating their significance and drawing a comparison with the preclinical emergence of the IFNa signature [29,85].

IL-21 is another important mediator in terms of B cells differentiation to plasma cells [86]. Animal models point to the essential role of this cytokine in the pathogenesis of SLE, as it has been found to be overexpressed in SLE models [86,87], leading to remission after blocking [88] and being linked with increased production of immunoglobulins. However, this particular cytokine not only participates in the pathogenetic pathway of SLE [89,90], but it also, due to the fact that it is produced by T follicular helper cells in B cell follicles [91], depicts disturbed interaction of T and B cells.

On the contrary, cytokines that regulate immune responses by mediating signals and enhancing the function of B regulatory cells (Bregs) seem to also be modified. It is interesting that higher expression and levels of Transforming growth factor-β (TGF-β), IL-10, and IL-35 were discovered in patients with newly diagnosed, untreated SLE, produced by Bregs and demonstrating in fact a negative correlation with increasing Systemic Lupus Erythematosus Disease Activity Index SLEDAI score [92]. In the case of IL-10, is it not quite clear how autoimmune diseases and especially SLE are associated, since other investigators showed significant aggravation of autoimmune diseases upon IL-10 absence [93]. It should be mentioned that IL-10 is not only produced by B regs but also by a wide range of immune cells of both the myeloid and lymphoid lineages, such as DCs, neutrophils, natural killer (NK) cells, CD4+, and CD8+ T cells [94], indicative of the complexity of the underlying mechanisms.

IL-35, an additional immunoregulatory cytokine that, in an autocrine manner, stimulates and is secreted by T- and Bregs [95], displays similar difficulties since various studies present controversial results [96,97]. This cytokine was examined in the subset of lupus nephritis, where elevated serum levels were discovered [98], while IL-35 related treatment showed amelioration of renal injury alongside with reduction of autoantibody levels [97].

IL-4 is a multifaceted agent and its contribution to SLE remains a topic of debate. IL-4 is produced by a slew of immune cells, among them activated CD4+ T cells, resulting in B cell class switching, favoring Th2 differentiation, inhibiting Th1 phenotype, and promoting the development of M2 macrophages, which are involved wound healing, tissue repair, and inflammation control [99]. A few studies indicate no significant differences in IL-4 expression between SLE patients and healthy controls, while others suggest that SLE is associated with reduced IL-4 production [100,101]. It is quite interesting that researchers found higher percentages of IL-4+/CD4+ T cells in the skin tissues of SLE patients compared to healthy individuals, highlighting IL-4’s role in local tissue lesions despite its relatively low serum levels [102]. It seems, however, that in SLE, IL-4 acts as a T-cell suppressor while its effect on B-cells is controversial, linked with B-cell activation and anti-DNA antibody production on the one hand and B cell inhibition on the other [103,104]. The interaction between IL-4 and B cells can result in reverting anergy in self-reactive B cells, promoting their activation and survival [105]. Despite ongoing controversy about IL-4 levels in SLE, it is clear that IL-4 plays a regulatory role in both humoral and cellular immunity.

#### 2.1.4. Autoantibodies Production

B cells perform a variety of functions that initiate and continuously channel T and B cell activation. The role of autoantibodies is doubtless. It is most obvious in the case of neonatal lupus erythematosus (NLE), where auto-antibodies, and not immune cells, pass through the placenta of a mother with SLE and can cause clinical syndrome to the neonate [106]. Moreover, autoantibodies can be found in the serum many years before the development of clinical syndrome [107], questioning their pathogenetic function. They cannot induce lupus events such as lupus nephritis on their own; additional conditions like insufficient immune complex clearance and T lymphocyte dysfunction must be met [108]. This should not, however, underestimate their position in the pathogenetic cycle. The main feature of lupus nephritis is the formation of ICs, most possibly in situ, located in glomeruli, launching an inflammatory process with the aid of complement and inflammatory cells but also intra-renal cells [109]. The most well studied fraction is anti-dsDNA, autoantibodies that turn against multi-targets, both extrarenal, including dsDNA, histones and chromatin, and renal antigens, such as α-actinin [110]. Apart from kidneys [111], autoantibodies are also located in skin lesions [112] or target neurons, causing Central Nervous System (CNS) manifestations [113].

Animal models proved the development of glomerulonephritis in MRL/lpr mice that were genetically altered to prevent them from secreting antibodies, but this did not apply in MRL/lpr mice that lacked B cells, indicating that B cells may not need the assistance of auto-antibody production to act as effector cells in SLE [33]. On top of this, B cell deficiency was associated with poor activation of T cells [114], emerging in their additional antigen-presenting role.

### 2.2. Changes in B Cell Phenotype Related to SLE

Phenotypic alterations in B lymphocyte compartment are described in Table 1.

#### 2.2.1. Expression of IgD and CD27 Molecules

Alterations in circulating B lymphocytes regularly reflect the pathogenesis and activity of autoimmune diseases [78,115,116,117]. Surface molecules that mainly determine B lymphocyte function and interaction with immune or inflammatory mediators are the clusters of differentiation (CD), which present as membrane receptors, at different stages of B cell differentiation. Their presence or absence may indicate certain and unique functional roles in immune surveillance, and also signify the activation, senescence, and exhaustion status of cells [118]. CD27 is a commonly used marker found on the surface of human memory B cells and becomes increasingly expressed during the germinal center reaction. For example, in humans, the majority of highly selected memory B cells display the classical memory marker CD27 on their surfaces, which belongs to the TNF superfamily. Therefore, CD27 is not only widely used as a marker for memory B cells but is also likely vital for B cell activation and memory B cell formation [119,120]. On the other hand, immunoglobulin D (IgD) on B cells plays several critical roles in regulating immune responses, mainly by reducing sensitivity to self-antigens of autoreactive B cells, preventing inappropriate immune activation. This balance between autoreactivity and foreign antigen responsiveness ensures immune tolerance [121].

In general, B lymphocytes are reduced in patients with active SLE. As has been described in extensive research conducted by our and other research teams, a shift of B cell population to advanced differentiated compartments, namely reduction of naïve (IgD+CD27−) and non-switched memory (IgD+CD27+) and increase in the proportion of switched memory (IgD-CD27+) and double negative (DN) (IgD-CD27−) B cells, is evident in SLE patients [122,123,124,125,126,127]. DN B cells consist of a heterogenous and bewildering B cell subset, which has recently attracted research interest as it seems to bring about extraordinary activities, such as in the ageing process, chronic inflammation, viral infections, and various types of cancer [128,129,130].

DN B cells are consistently elevated in SLE regardless of the disease’s activity status, and for many years there has been a debate whether this is part of the immune activation cascade in SLE or simply the result of sustained chronic inflammation. It is clearly demonstrated that in a complex systemic autoimmune disease such as SLE, the immune reactions are extremely difficult to separate as cause or effect. However, the latest research reveals that DN B cells in SLE patients are mainly characterized by the lack of C-X-C chemokine receptor type 5 (CXCR5), the follicular homing marker, characterized by CD19+IgD-CD27-CXCR5-, defined as DN2 B cells, and seem to hold an immunopathogenic role in the onset of disease. DN B cells may arise from defective germinal center reactions or originate from memory B cells following the displacement or downregulation of the CD27 molecule during immune senescence or exhaustion. In SLE, the increase in DN2 B cells may be the result of hyperresponsiveness to TLR-7 signaling [131,132].

#### 2.2.2. Age Associated B Cells

Age Associated B cells (ABCs) are described as a heterogeneous B lymphocyte subpopulation, characterized by the expression of T-bet and defined as CD19+CD21-CD11c+T-bet+. T-bet transcription factor is expressed in various immune cells, including T helper 1 cells (Th1), NK cells, DCs, and B cells, and plays a crucial role in coordinating their activity status. In B lymphocytes, T-bet expression is associated with memory B cells, where it is gradually upregulated as a result of synergistic stimulation of specific BCRs, TLR7, and either the IFN-γ or IL-21 receptor [133].

Although the phenotypic characteristics of ABCs are precisely described and widely accepted, their functional features and possible correlation with aging and chronic inflammation are still under investigation. As their population gradually increases in elderly people, these cells are often considered as markers of immunosenescence. T-bet expression is upregulated in chronic systemic inflammatory diseases, such as SLE, rheumatoid arthritis, Crohn’s disease, Multiple Sclerosis, and celiac disease. T-bet plays a significant role in various aspects of autoimmunity, including formation of spontaneous germinal centers, enhanced antigen presentation to T cells, and production of autoreactive IgG, acting as precursors of autoantibody secreting cells [134,135].

Experimental models of SLE have proved the upregulation of T-bet expression following stimulation with IFN-γ but not IFN-α, revealing its critical role in the onset of autoimmune response, the association with lupus-like syndrome, and the beneficial effect of eliminating T-bet expression. More precisely, T-bet deletion resulted in improved kidney function, ameliorated histological damage, and improved survival [134,136,137].

At a clinical level, noteworthy findings have been observed. CD11chiT-bet+ B cells appear to be linked to the percentages of various anti-nuclear autoantibodies associated with SLE (such as dsDNA, nucleosome, RNP, Smith, etc.), though it is highly likely that other B cell populations may also exhibit significant correlations. Additionally, these cells are more prevalent in SLE patients compared to healthy individuals, with their increase being associated with the severity of clinical symptoms. Specifically, the frequency of these cells correlates with a patient’s SLEDAI score [122,138]. Even more interestingly, DN2 B lymphocytes, which also express T-bet are potentially the most prominent cell types in SLE patients, and their expression and function are still under investigation [139].

Our recent research on peripheral blood DN B cells revealed their increase in patients with SLE, independent of disease activity, and, more interestingly, a significant positive correlation with early differentiated CD4+ and CD8+ lymphocyte subpopulations. All the subtypes tested, including CD4+CD45RA+CD28+, CD4+CD45RA+CD57−, CD4+CD45RA-CD57−, CD4+CD28+CD57−, CD4+CD28+CD57+, CD4+CM, CD8+CD31+, CD8+Naive, CD8+CD45RA-CD57−, CD8+CD28+CD57−, and CD8+CD28+CD57+, correlated with DN B cells in SLE patients tested. However, multiple regression analysis revealed that CD4+CD31+, CD8+CD45RA-CD57−, and CD8+CD28+CD57− cells were independent subtypes contributing to DN B cell population [123,125].

These findings cannot delineate whether T and B lymphocytes are spontaneously activated by a common pathogenic mechanism as part of autoimmune dysregulation or whether DN B cells, antigen presenting cells, are implicated in the stimulation of peripheral T lymphocytes. However, they prove that DN B lymphocytes play a major role in the alterations of adaptive immunity in SLE.

#### 2.2.3. B Regulatory Cells

Bregs stands for a specific subset of B lymphocytes charged with immunomodulatory-immunosuppressive functions through IL-10 secretion and other cytokines as well as intercellular communication [140]. It has been documented that during immunological responses, different types of B cells, namely naive B cells, immature B cells, and plasma cells, can all develop into Bregs in response to environmental stimuli [141]. We are unable to identify a single phenotypic combination that characterizes these cells though, as many animal models indicate that Bregs, which produce immunoregulatory cytokines such as IL-10, IL-35 etc., are derived from distinct B cell subtypes [93,142,143,144]. In any case, most of the existing studies exhibit an expansion of Breg cells in peripheral blood of lupus patients [145,146,147,148], sometimes exhibiting correlation with SLE disease activity. This upregulation was suggested by one study to be a feedback mechanism after a T follicular helper (Tfh) increase [146]. However, possibly due to different surface molecule characterization, controversial data show a reduction of this subset, especially in those with renal disease [149,150,151]. This discordance is also evident in experimental studies [152,153], establishing an unsolved issue.

Regardless of the amount of Bregs, the functionality of these cells tends also to reduce. Initial pDC stimulation of immature B cells that encourages their evolution into Bregs is the first step in the process. There is evidence that in SLE, this process is impaired and eventually results in favor of plasmablast differentiation. Bregs, in addition to restraining the production of IFN-α from pDCs through IL-10 release, act under normal circumstances to suppress T helper cells and prohibit the production of cytokines such as TNF-α and IFN-γ, an ability that Bregs from lupus patients seem to lack [150,154]. In addition, the protective role of Bregs was demonstrated experimentally, as the transfer of such cells can lead to deceleration of SLE progression in MRL/Lpr lupus-prone mice [155]. Consequently, in SLE, there is a distinct dysregulation of Bregs functionality.

## 3. T Lymphocytes Related Abnormalities

**Figure 2:**
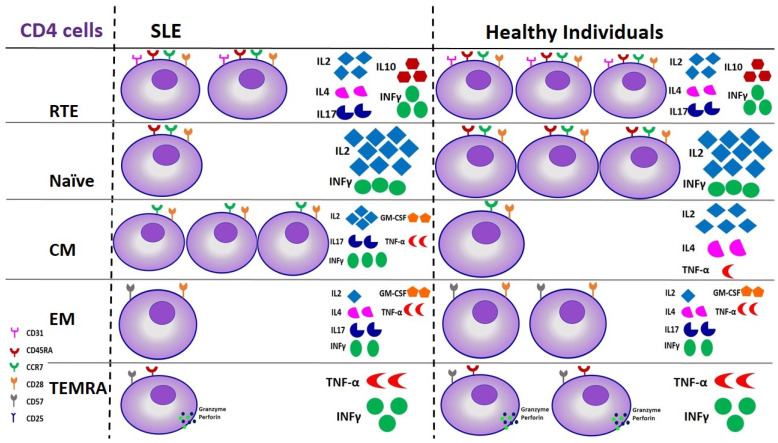
Derangements of CD4 lymphocyte subsets in SLE patients compared to healthy individuals in terms of cell number and function. The figure depicts a 3-fold upregulation of CM cells in patients with SLE, followed by changes in cytokine production, and a subsequent 2/3 reduction of RTEs, 1/3 of Naïve, and 1/2 of EM and TEMRA cells, which, however, are not followed by alterations in their cytokine production. SLE: Systemic Lupus Erythematosus, RTE: Recent Thymic Emigrants, CM: Central Memory cells, EM: Effector Memory cells, TEMRA: Terminally differentiated cells.

**Figure 3:**
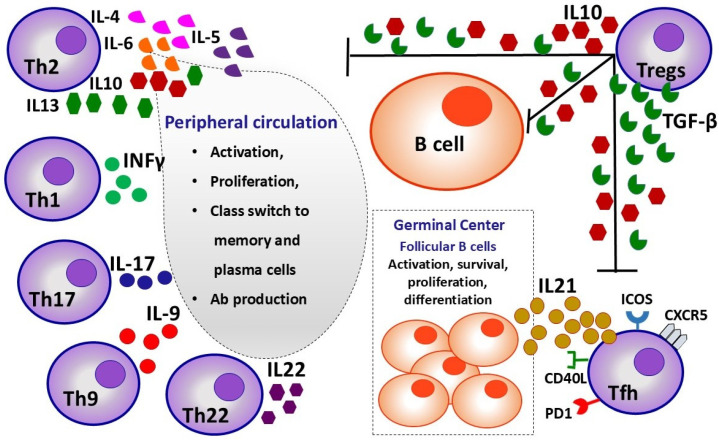
Influence of proliferation, differentiation, and activity of Th lymphocyte subpopulations on B lymphocytes in germinal centers and peripheral circulation.

For many years, T lymphocytes were regarded as the principal players in the pathogenesis of SLE; therefore, treatment strategies aimed toward cellular immunity and cytokine production. Nowadays, we believe that T lymphocytes engender their effects mainly through the interaction with B cells and production of pre-inflammatory cytokines [3,156]. The threshold of T lymphocyte activation is reduced, leading to their immoderate activation, as this becomes evident by a clear shift of T lymphocyte subpopulations to activated subtypes, such as Central Memory (CM) cells and autoreactive memory T cells, and increased production and secretion of pre-inflammatory substances, cytokines, and growth factors [157,158,159,160], as depicted in Figure 2 and Figure 3.

Furthermore, Regulatory (Tregs) and cytotoxic T cells are reduced, changes that further support T cell activation, as their depletion allows the expansion of pro-inflammatory and Tfh cell subpopulations. These subtypes are able to initiate inflammation, as they infiltrate tissues and stimulate peripheral B lymphocytes in the auto-antibody production [30,161,162].

### 3.1. CD4 Lymphocytes

The role of CD4+ lymphocytes in SLE is seemingly crucial, involving concomitant and collaborative mechanisms that synergistically lead to disease initiation. Such mechanisms encompass reduction and dysfunction of Tregs, dysregulation of follicular T cells, reduction of naïve CD4+ cells, and concomitant expansion of CM and exhausted CD4+ cells, while changes in the expression of membrane or cytoplasmic indices, increase of Inducible costimulator (ICOS), phosphatase 2A, NF-kB, Rho-associated coiled-coil domain protein kinase (ROCK), mTOR, etc. will activate cell signaling pathways [123,162,163,164,165].

Alterations of CD4 lymphocyte subsets are depicted in Figure 2.

#### 3.1.1. T Helper 1 Lymphocytes

Th1 cells derive from Naïve CD4 cells following stimulation of transcription factor T-bet by IL-12, and they are involved in the defense against intracellular pathogens, such as bacteria and viruses, and in the instigation of inflammatory responses, mainly responsible for producing IFN-γ, a cytokine crucial for cellular immunity. Through these characteristics and activities, Th1 cells play a crucial role in orchestrating inflammatory and immune responses in several systemic immune diseases, such as SLE. Although SLE is traditionally considered as a Th2 associated disease, because of the increased autoantibody production by activated B lymphocytes, the role of Th1 cells in the initiation and escalation of immune and inflammatory reactions cannot be ignored.

Th1 cells seem to be involved in disease pathogenesis, mainly through the production of IFN-γ, the cytokine that holds a key role in initiating and maintaining systemic inflammatory reactions. IFN-γ plays multiple roles, such as promoting macrophage and antigen presenting cell activation, increasing B cell responsiveness to IL-21, inducing autoantibody production by activated B lymphocytes, and participating in tissue inflammation through facilitating immune complex deposition and complement activation. Several studies have proved a close association between serum levels of IFN-γ and severity of SLE. Increased IFN-γ levels may induce expansion of the T-bet expressing DN2 cells, while T-bet^hi^ pre-ASCs can derive from naïve B cells after being stimulated by Th1 cells or IFN-γ in collaboration with IL2 and-TLR7/8 ligands [166,167]. Increased activity and upregulation of Th1 cells are also associated with the presence of lupus nephritis. Lipocalin-2 may stimulate the IL-12/STAT4 signaling pathway, which promotes upregulation of IFN-γ expression on CD4 cells and their differentiation into Th1 cells and leads to kidney involvement in SLE [168].

There are also reports that support a protective role for Th1 in SLE, as they seem to antagonize Th17 by suppressing their excessive responses [169]. Moreover, most of the conventional treatment modalities in SLE aim to increase ratio of Th1/Th2 cells, and the results from a recent meta-analysis have proved that patients who are under treatment have increased Th1 concentrations compared to untreated patients [170].

#### 3.1.2. T Helper 2 Lymphocytes

Th2 cells have a central and crucial role in the pathogenesis and progression of SLE through their capability to produce cytokines that direct B cell differentiation into plasma cells and govern production of autoantibodies [171,172]. Under normal conditions, Th2 cells are responsible of mediating the immune response against extracellular pathogens, such as parasites and promoting antibody-production [173,174]. Th2 cells are primarily associated with the production of anti-inflammatory cytokines IL-4, IL-5, IL-10, and IL-13; all of them promote B cell class switching to plasma cells and also encourage M2, instead of M1, macrophage formation, which are involved in epithelial cell regeneration and tissue repair and eliminate tissue inflammatory damage; they also increase collagen production and lead to fibrosis [175,176].

Differentiation of B lymphocytes into plasma cells and increased immunoglobulin secretion may turn into autoantibody production and contribute to the pathogenesis of SLE, with increased levels of cytokines playing complementary roles, such as IL-4 cytokine promoting B cell differentiation into auto-antibody producing cells, IL-13 maintaining B cell survival, and IL-5 supporting eosinophil survival and tissue inflammation. The imbalance in Th1/Th2 ratio favors Th2 immune responses. Th2 cytokines contribute to loss of tolerance to self-antigens, while suppressed Th1 mediated immunity leads to impaired capability to clear immune complexes and cellular debris.

There is much controversy between researchers regarding this balance, and it is possibly affected by gender and age, as Th2 cells are more pronounced in older and male patients [170,177]. The exact mechanism, however, that guides CD4 lymphocyte differentiation towards Th2 cells is not yet fully investigated. Activated Naïve B lymphocytes in SLE patients are capable of upregulating T-cell costimulatory molecules, and also endorse CD4^+^ cell proliferation and polarization to Th2 and Th17 cells. In a paracrine way, Th2 cell signals may force naïve B cell differentiation into autoantibody secreting cells producing inflammatory cytokines and anti-DNA antibodies [178]. Overproduction of IL-17 and simultaneously reduced production of IL-2 by Th2 cells in patients with SLE leads to dysfunction of regulatory T cells and their inability to restrain inflammation.

A cascade of early signaling defects, along with aberrant activation of kinase signaling pathways, result in altered lymphocyte phenotypes by disrupting the metabolic profile and epigenetic landscape. In vitro studies proved that Th1, Th2, and Th17 cells from patients with SLE present increased rates of apoptosis compared to healthy controls [179]. Furthermore, in vivo studies have shown that all major metabolic pathways, including glycolysis, glutaminolysis, and oxidative phosphorylation, are altered in T cells from lupus-prone mice, while similar defects are evident in patients with SLE [180].

#### 3.1.3. Regulatory T Lymphocytes

Regulatory T cells (Tregs) are CD4 subsets, characterized by the presence of CD25 and FoxP3, and play a critical role in modulating immune responses through the inhibition of proliferation and activation of B lymphocytes and CD4+ cells and by impeding the differentiation of cytotoxic CD8+ T cells. Over the years, numerous studies have investigated Treg cell population and function in SLE, and most of them report reduced numbers or impaired function of circulating Tregs. This depletion contributes to T cell activation by giving space to the expansion of pro-inflammatory and follicular helper T cell subpopulations, which in turn act by initiating inflammation, as they are able to infiltrate tissues and stimulate peripheral B lymphocytes to auto-antibody production [30,161,181]. Several mechanisms have been described for the reduction of Tregs in SLE; most recently, interest has been focused on the glycosylation process of T cell receptors and co-receptors. In fact, impaired interaction between CD69 and the S100A8/S100A9 complex, caused by reduced sialic acid on CD4 cells, will directly affect Treg cell differentiation [182]. Moreover, increased expression of Glucocorticoid-induced TNFR-related protein (GITR), programmed death-1 (PD-1), PD-L1, CD73, Cytotoxic T-lymphocyte associated protein 4 (CTLA-4), and ICOS on Τregs seems to down-regulate their suppressive capacity [183].

However, there are few investigators who found no significant abnormalities or even elevated levels of Tregs in SLE patients compared to healthy controls; this supports the notion that increased T lymphocyte activity is attributed to resistance of the effector T cells to T regulatory-mediated suppression rather than to a numeric or functional defect in the Tregs themselves. Although these conflicting results may sometimes be the result of technical issues, such as variations in flow cytometry staining protocols and isolation techniques, the population of Tregs may also be influenced by the status of the disease [184,185].

IL-10, as mentioned earlier, is an anti-inflammatory cytokine produced by a variety of immune cells, one of which are Tregs. Alongside the many pathways that this cytokine interferes with, including the procedures of antigen-presenting cells (APCs) [186], it also amplifies proliferation and differentiation of B cells. This might sound controversial but, as in any other case, the microenvironment tends to play an important role. In SLE, things seem to be simpler, as studies found elevated levels of IL-10 in peripheral blood and tissue-targets [187,188], and it seems to be related to the pathogenesis of the disease as its experimental blockage led to decreased autoantibody secretion [189].

##### Interactions of Regulatory T Cells with Other T Lymphocyte Subtypes

The regulation effect of Tregs on immune cells, particularly their effect on activation and proliferation of other T lymphocyte subsets, is multifaceted, interactive, and multilayered, and has been extensively studied. In vitro experiments have proved that the key mediators in such suppressive activity are the Treg production of anti-inflammatory cytokines, IL-10 and TGF-β, as well as the expression of co-stimulatory molecules on the T cell surface.

In vitro and in vivo experiments have shown that Tregs may reduce Th1 proliferation and IFN-γ cytokine production. These reactions involve IL-2 signaling, TGF-β production from Tregs, and CTLA-4 and PD-1 expression on Th1 cells and are evident even in the presence of IL-12 [190,191]. Tregs, through the secretion of IL-10, which antagonizes IL-4 activities, are capable of suppressing IL-4 production and Th2 cell differentiation. The expression of FoxP3 seems to play a key role in Treg/Th2 interaction, as downregulation of FoxP3 expression in Tregs was followed by a strong and selective increase of IL-4, IL-5, and IL-3, leading to increased Th2 differentiation [192,193].

Interactions between Tregs and Th17 cells are more complicated, as both cell types respond to IL-2, IL-10, TGF-β, and CTLA-4. In vitro experiments demonstrated a direct effect of Tregs in Th17 differentiation in the presence of TGF-β. Other studies have shown the beneficial effect of neutralizing IL-6 in the suppression of Th17 cells by Tregs. Even more interestingly, Tregs that have lost the FoxP3 molecule may convert into Th17 after under pro-inflammatory conditions [194,195,196].

There is a certain competition between Tregs and Tfh cells’ functions. Regulatory T cells may repress Tfh cell differentiation and their IL-21 production through direct cell-cell contact mechanisms through the secretion of IL-10, but also involving CTLA-4 and ICOS-L molecules. In vitro studies have proved that follicular regulatory T (Tfr) cells that express CXCR5, a marker that allows them to localize to follicular regions, are particularly effective at suppressing Tfh cell activity. However, their suppressive effects on the B cell stimulation by Tfh cells was restricted to B cell proliferation and class switch recombination and had no substantial effect on activated B lymphocytes. IL-4 was the main contributor in these effects while IL-21 had only a minimal role [197,198]. Recent studies show that inhibition of IL-21 by blocking its receptor with an anti-IL-21R monoclonal antibody resulted in plasmablast generation and immunoglobulin production [199].

##### The Role of FoxP3 Isoforms in the Function of Regulatory T Cells

The main and central role of the Regulatory T cell immunoregulatory function is attributed to FoxP3 expression. The FoxP3 gene in humans is alternatively spliced to encode different isoforms, the predominant ones being the full-length isoform (FL FoxP3) and the isoform lacking exon 2 (ΔE2 FOXP3); less frequently, ΔE7 FOXP3 and ΔE2ΔE7 FOXP3 isoforma are described. Exon 2 encodes a region important for the interaction with nuclear factor of activated T-cells (NFAT) and activator protein-1 (AP-1). Due to the lack of exon 2 and consequent impaired interaction with NFAT, as well as reduced production of IL-10 and TGF-β, the ΔE2 FOXP3 isoform is characterized by reduced suppressive activity of Th1, Th17, and Tfh cells. Even more interestingly, under certain inflammatory situations, these cells may produce IL-17 and behave as Th17 cells [200].

In cell culture models, ΔE2 FOXP3 Tregs are unstable, probably due to lower CD25 expression, and less effective, with reduced ability to suppress effector T cell subsets, particularly Th1 and Th17 cells, and could induce autoimmunity when transferred into *Tcrb*-deficient mice [201]. In vivo studies have shown that the presence of ΔE2 FOXP3 in mice was followed by excessive Tfh activity, leading to increased GC B lymphocyte response, production of anti-dsDNA and antinuclear autoantibody production, and also immune complex glomerulonephritis [202].

Human studies evaluating the FoxP3 isoforms are still limited. Reduction of circulating TGF-β leading to impaired immunoregulation has been proved in active SLE. The exact role of ΔΕ2, ΔΕ7, and ΔΕ2ΔΕ7 isoforms, however, need further evaluation in human SLE, as the expression of both FL-and ΔE2 isoforms were reduced in patients with SLE and rheumatoid arthritis [203,204,205].

The Glycoprotein A Repetitions Predominant (GARP) and Latency-associated peptide (LAP) axis is essential for the activation of TGF-β and the suppressive function of Tregs. GARP is a transmembrane receptor expressed on activated Tregs, which binds to latent TGF-β and is crucial for activating TGF-β. LAP is a component of the latent TGF-β complex, which prevents TGF-β activation until it is free. Recent studies have proved the importance of surface GARP-TGF-β as a checkpoint that regulates B cell peripheral tolerance, leading to autoimmune disease pathogenesis [206]. Disruption of this pathway in vitro leads to reduced Treg-mediated anti-inflammatory function [207,208].

#### 3.1.4. T Helper 22 Lymphocytes

Th22 cells represent a distinct cell lineage, derived from Naïve CD4 cells, under the guidance of whole cascade of cytokines and signaling pathways, which are implicated in their development, proliferation, and function. IL-6 and TNF-α play certain role in Th22 differentiation, while IL-23 supports their survival and expansion. The presence of certain transcription factors, such as aryl hydrocarbon receptor, characterizes their unique function [209].

Th22 exclusively produces IL-22, a function that differentiates it from Th-17 cells. Apart from the aryl hydrocarbon receptor as the key transcription factor, it also expresses the CCR4 and CCR10 skin-homing receptors and the CCR6 chemokine receptor. In healthy individuals, it participates in mucosal defense, tissue repair, and wound healing [209,210].

IL-22, a proinflamatory cytokine from the IL-10 family, is produced mainly by Th22 cells, but also by other immune cells including γδ T cells, NK cells, NK T cells, and mucosal associated invariant T cells. IL-22 mediates systemic and local inflammatory responses, limiting tissue inflammation. There are reports, however, that are suggestive of a dual function, anti-inflammatory and pro-inflammatory, depending on the surrounding microenvironment. Through binding to its receptor, leading to the formation of the IL-22-IL-22R1-IL10R2 complex, IL-22 promotes phosphorylation of STAT molecules (STAT3, STAT 1, STAT5) and activation of MAPK signaling pathways, including MEKERK-RSK, JNK/SAPK, and p38 kinase, and the PI3K-Akt-mTOR pathway in epithelial cells and keratinocytes [211,212,213].

Th22 cells and IL-22 cytokine have been implicated in the pathogenesis of several autoimmune diseases, including psoriasis, autoimmune hepatitis, immune thrombocytopenia, rheumatoid arthritis, and SLE [214]. Increased proportion of peripheral Th22 and Th17 cells have been found in patients with SLE when compared to healthy individuals, together with concentrations of IL-22, IFN-γ, TNF-α, and IL-17 cytokines. More interestingly, plasma IL-22, Th22, and CCR6+Th22 cells were increased in patients with newly diagnosed lupus nephritis and had significant correlation with disease activity [213,215].

However, these findings were not confirmed by other studies, and a lot of debate persists in the literature regarding the exact role of Th22 cells and IL-22 cytokine, especially in the pathogenesis and activity of SLE. Several investigators have described an inverse correlation between Th22 concentration and disease activity, while recent in vitro experiments showed that α-IgM- and α-CD40-activated B cells could reduce Th17 and increase Th22 cell differentiation, resulting in a reduction in the apoptotic rate of renal endothelial cells. In vivo experiments proved that injection of activated B cells in MRL/lpr mice could promote Th22 cell differentiation and IL-22 cytokine production, reduce Th17, as well as diminish dsDNA antibody titer and proteinuria and alleviate lupus nephritis [216]. All these discrepancies in the concentration of Th22 cells and their role in disease pathogenesis and activity can possibly be attributed to differences in timing of evaluation, organ involvement, and medication used. Most investigators, however, associate dysregulation of Th22 cells with the severity of skin lesions and kidney involvement in SLE. In fact, different IL-22 levels have been described in the presence of an initial diagnosis or relapse of lupus nephritis and even in the presence of different histologic subtypes [217].

#### 3.1.5. T Follicular Helper Lymphocytes

Tfh cells constitute a specific subgroup of CD4+ T cells that mainly mediate the selection of high-affinity B cells in germinal centers [218]. These cells are characterized by the expression of certain molecules, namely the transcription factor BCL6, C-X-C chemokine receptor type 5 (CXCR5), which enables them to migrate into germinal centers, PD-1, ICOS, and CD40L. The main IL that they secrete is IL-21, a cytokine necessary for effector functions [219]. They classically reside in secondary lymphoid organs; however, T cells with similar markers can be detected in the blood, and are supposed to represent a “memory” compartment that can be used in case of need [220]. Most human studies focus on the investigation of this circulating subset, the circulating Tfh (cTfh) cells, as it is more approachable. In SLE, they were found to be expanded and in close association with disease activity and with the levels of serum autoantibodies [164,221,222]. Further research regarding subcategorization of cTfh into cTfh1, cTfh2, and cTfh17 demonstrated controversial results among various studies [163,199,223]. Moreover, a discordance exists regarding the presence or absence of nephritis [199], where cTfh17 proportion shows a decrease in and relation with disease activity. Of great interest is the fact that working groups have detected Tfh cells inside renal tissue in lupus nephritis patients [224].

#### 3.1.6. T Helper 9 Lymphocytes

Effector Th9 cells derive from naïve T helper cells under stimulation of IL-9 cytokine and the influence of regulatory factors, TGF-β, IL-4, and certain environmental signals. Initially classified as Th2 cells, they have only recently been recognized as a distinct subset characterized by the production of Interferon Regulatory Factor 4 (IRF4) and IL-9 cytokine. IL-9 cytokine makes an important contribution to allergic reactions; it stimulates eosinophil and mast cell proliferation and recruitment and is implicated in the pathogenesis of allergic diseases, such as rhinitis, asthma, and atopic and contact dermatitis, but also to autoimmune diseases, including SLE, RA, psoriasis, and inflammatory bowel disease. IL-9 is implicated in the proliferation of several CD4+ subpopulations, and therefore its contribution to SLE pathogenesis seems possible. There is, however, evidence of anti-inflammatory functions and regulation of immune responses [225,226].

In vivo studies have demonstrated increased IL-9+ lymphocytes in the spleen and kidneys of lupus-prone mice and significant correlation with GC cells. Moreover, increased circulating IL-9 levels had a close association with anti-ds DNA antibodies. It seems possible that Th9 cells can promote B cell proliferation and switch to plasma cells and stimulate autoantibody production. Administration of IL-9 neutralizing antibodies reduced serum anti-dsDNA-antibody titers and alleviated lupus nephritis in MRL/lpr mice [227,228].

#### 3.1.7. T Helper 17 Lymphocytes

Most importantly, Th17 (T helper 17 lymphocytes) is the T lymphocyte subtype that probably plays the key role in SLE and predominates in disease activity and tissue damage. Generation of Th17 cells and their expansion in SLE or other autoimmune diseases has attracted research interest, and recent studies have shown that specific species of the microbiota grow selectively, and, under certain situations, may involve innate and adaptive immunity and activate different T-cell subsets by using complicated mechanisms [229]. Close correlation between gut microbiota and their metabolites with the regulation of T lymphocytes has been widely accepted [230]. There is accumulating evidence that Th17 cells derive from the gut, following the activity of gut microbiota, especially adhesive bacteria including segmented filamentous bacteria [231,232,233]. Segmented filamentous bacteria (SFB) induce Th17 cell differentiation, while the disruption between SFB and *Bacteroides fragilis clostridia* species will disrupt the homeostasis between Th17 and Tregs and result in the development of Th17-mediated autoimmune diseases [234,235].

RORγt, C-C-motif chemokine receptor 6 (CCR6), IL-23 receptor, IL-17A, and IL-17F are characteristic of Th17 cells, which infiltrate tissues and cause damage by the release of IL-17A and IL-17F cytokines, causing further inflammation and damage [236].

Mechanisms implicated in the alterations of Th17 cells are not completely delineated; they include an adverse relation to Tregs regulation and involve a whole cohort of cytokine interaction [237,238]. A recent meta-analysis of 35 studies revealed increased levels of Th17 cells and increased ratio of Th17/Tregs, followed by increased serum concentration of IL-17, IL-6, IL-21, and IL-10 but reduced TGF-β1 levels. Th17 cells and high IL-17 and IL-6 levels were found to have significant correlation with disease activity, and also with age, gender, and treatment regimes. Tregs were reduced in patients with SLE, although sub-group analysis showed that reduced levels of Treg cells and an increased ratio of Th17/Tregs was more prominent in elderly patients and those with active disease, while previous investigators suggested that increased ratio of Th17/Tregs was found in males and had a close correlation with the development of atherosclerosis [239,240].

Growth factor TGF-β has a central role in the development of both Th17 and Tregs. The difference in this process depends on the type of cytokine preponderance. Interaction of TGF-β with IL-6 will promote the development of Th17 cells, especially in the presence of increased IFN-a, while the same TGF-β, when it collaborates with IL-2, will result in the upregulation of Tregs. Therefore, in SLE patients, increased production of IFN-a by pDCs stimulates myeloid cells to synthesize and excrete IL-6, facilitating the development of Th17 cells, impairing the suppressive effect of Tregs, but most importantly forcing the transformation of Tregs into Th17 cells. In addition, the simultaneous reduction of IL-2 also participates in the reduction of Tregs to the benefit of Th17 cells [241,242].

The multiple interactions between all subpopulations of T helper cells and B cells are described in Figure 3.

### 3.2. CD8+ T Lymphocytes

**Figure 4:**
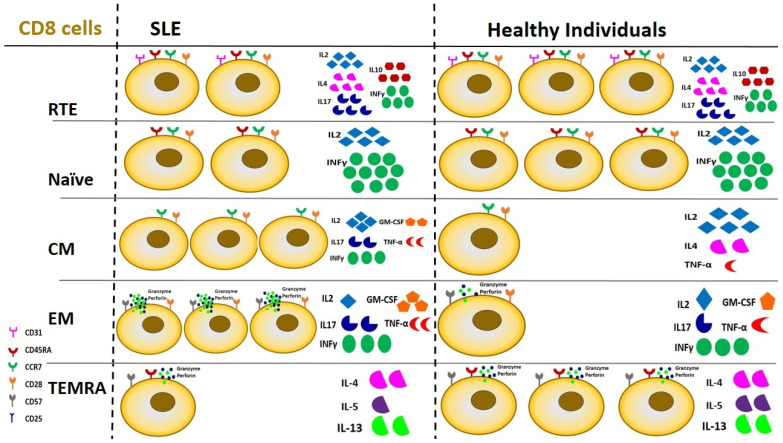
Derangements of CD8 lymphocyte subsets in SLE patients compared to healthy individuals in terms of cell number and function. CM and EM CD8 cells demonstrate a 3-fold upregulation in patients with SLE, followed by certain changes in cytokine production. RTE and Naïve cells are reduced by 2/3 and TEMRA cells by 1/3 compared to healthy individuals, with no significant changes in cytokine production. SLE: Systemic Lupus Erythematosus, RTE: Recent Thymic Emigrants, CM: Central Memory cells, EM: Effector Memory cells, TEMRA: Terminally differentiated cells.

Regarding CD8 cells, a clear preponderance of CM subtypes was evident in a recent study in patients with lupus nephritis; at the same time, the exhaustion process predominated among peripheral CD8 cells, indicating both their persistent and chronic activation [123,157]. Figure 4 elucidates differences in CD8 lymphocyte subpopulations between SLE patients and healthy individuals regarding peripheral cell number and cytokine production.

Activated CD8 cells, together with naïve CD4 cells, had significant correlation with DN B cells, supporting a possible role of early differentiated and activated T cells in the dysregulation of B lymphocytes, probably through their response to TLR-7 signaling [137]. CD8 lymphocytes in SLE patients are characterized by reduced response to viral infections, and this has been attributed to the reduction of Signaling lymphocytic activation molecule family member 4 (SLAMF4) expressed on memory CD8+ cells, increased expression of PD-1 CD8 cells, and transformation of CD8+ to Double-Negative T cells (DN T cells) [243,244]. Another interesting finding is the presence of these cells in urine samples from patients with lupus nephritis [245].

### 3.3. γδT Cells

T cell development begin in the thymus, where T cells pass through a series of developmental stages, starting from double negative (CD4−CD8−, DN), double positive (CD4+CD8+, DP), and single positive (CD4−CD8+ or CD4+CD8−, SP) cells. Based on the subsequent expression of CD44 and CD25 molecules, DN T cells are divided into four discrete stages: DN1, 2, 3, and 4. Differentiation to αβ, representing the vast majority of T cells, or γδ T cells accounts for less than 5% of peripheral T cell population, and occurs during the DN2b and DN3a stages [246]. Although αβ and γδ T cells have common characteristics and functions, including production of cytokines, activation and class-switching of B cells, and cytotoxic activities, their main difference consists in their connection to major histocompatibility complex (MHC) molecules. TCRs on γδT cells have a very limited repertoire; they do not recognize MHC binding molecules, but instead these cells bind to non-peptide substances, antigens from bacteria, fungi and parasites, t-RNA synthetases, and glycosides. In vitro studies have shown that γδT cells can activate B lymphocytes through the upregulated expression of CD40 and promote immunoglobulin production [247].

The population of peripheral γδT cells in SLE patients and their role in disease pathogenesis has been a subject of debate between investigators, as there are reports of both increased population in SLE and findings of reduced numbers in newly diagnosed active disease [248,249]. Most researchers, however, agree that *γδ*T cells are increased on affected tissues, such as skin, and are associated with disease activity [250]. Furthermore, γδT cells in SLE patients produce large amounts of IFN-*γ*, TNF-*α*, IL-10, IL-4, and IL21. It is possible that subsets of γδT cells have different functions, resembling either Th1, Th2, Th17, or Treg cells, and can also divert from one phenotype to another, exerting cytotoxic or immunoregulating effects [251,252].

### 3.4. Double Negative T Lymphocytes

DN T cells are expanded in SLE patients, and accumulated evidence supports their critical role in the pathogenesis of the disease. DN T cells are defined as TCRαβ+CD4−CD8−, and can derive from late stage double negative thymocytes that migrate to intestinal epithelium or from exhausted peripheral CD8 cells following continuous stimulation [253]. Supporting this option, recent studies proved a link between loss of splenic marginal zone macrophages and generation of DN T cells. Investigators showed that loss of splenic marginal zone macrophages, cells that are responsible for immune tolerance, will lead to impaired tolerogenic clearance of apoptotic cell blebs and release of self-antigens followed by a cytokine cascade, which facilitates the generation of DN T cells after sustained activation of self-reactive CD8^+^ T cells [254]. In vitro studies have shown that DN T cells proliferate after stimulation by anti-CD3 Ab and produce significant amounts of IL-17 and IFN-γ. An increased production of IL-17 and IL-23 cytokines in SLE patients seems to be due to the excessive production of CD4+ T cells and expanded DN T cells [255]. Sjogren’s syndrome, lymphoproliferative syndrome, and aplastic anemia have also been associated with impaired apoptotic cell clearance procedure and are associated with expansion of DN T cells [256,257,258].

DN T cells have a pro-inflammatory phenotype, characterized by an increased ability to produce IL-17, to migrate into tissues, and to assist B cells to produce autoantibodies. In addition, DN T cells from patients with SLE are also characterized by acquired proliferating features, including Ki67 expression, diluted TREC, and narrowed TCR repertoire. Most important is their tissue migration ability, which has been confirmed by their presence in kidneys of patients with lupus nephritis, where they produce the inflammatory cytokines IL-17, IFN-γ, and IL-4. In addition to this, they cause mitochondrial dysfunction, mammalian target of rapamycin (mTOR) activation, and B cell stimulation and contribute to the pathogenesis of kidney damage in patients with SLE [255,259].

## 4. Conclusions

The pathogenesis of SLE is profoundly influenced by the dysregulation of B and T lymphocytes, which play essential roles in the breakdown of self-tolerance and the progression of autoimmunity. B cell abnormalities, such as loss of self-tolerance, overexpression of B cell activation factors, and disruptions in B cell receptor and Toll-like receptor signaling, result in the uncontrolled generation of autoantibodies and cytokines, which fuel disease activity. Concurrently, T lymphocytes’ maladaptive responses, particularly the upregulation of pathogenic Th17 cells and the loss of regulatory T cells, aggravate immune-mediated tissue damage. In the case of kidney involvement, major dysregulations affect not only extra but also intrarenal lymphocyte processes. The interaction of these dysregulated immune cells displays the complexity of SLE and emphasizes the importance of targeted therapeutic strategies that address the individual immunological dysfunctions. Future research should focus on elucidating the precise molecular mechanisms governing B and T cell interactions in SLE, aiming to develop precision-based therapeutic strategies that can mitigate disease progression and improve patient outcomes.

## Figures and Tables

**Table 1:** Dysregulation of peripheral B lymphocyte subpopulations in patients with SLE, compared to healthy individuals.

Phenotypic Alterations of B Lymphocytes in SLE	
Upregulation	Downregulation
Switched Memory (CD19+IgD-CD27+)	Naïve (CD19+IgD+CD27-)
Double Negative (CD19+IgD-CD27-)	Non-Switched Memory (CD19+IgD+CD27+)
Age Associated B cells (CD19+CD21-CD11c+Tbet+)	
B regulatory cells (secreting IL10, IL5)	

## Data Availability

Research data are available upon request.

## Abbreviations

ABCs	Age Associated B cells
ANA	Antinuclear antibodies
AP-1	Activator protein-1
APCs	Antigen-presenting cells
APRIL	A proliferative-inducing ligand
ASC	Antibody-secreting cells
asN	Activated naïve B cells
BAFF-Rs	B-cell activating factor receptors
BCMA	B cell maturation antigen
BCRs	B-cell receptors
BLys	B lymphocyte stimulator
Bregs	B regulatory cells
CCR6	C-C-motif chemokine receptor 6
CD	Clusters of differentiation
CM	Central memory
CNS	Central Nervous System
cTfh	Circulating Tfh
CTLA-4	Cytotoxic T-lymphocyte associated protein 4
CXCR5	C-X-C chemokine receptor type 5
DCs	Dendritic cells
DN	Double negative
DN T cells	Double-Negative T cells
EF	Extrafollicular
GARP	Glycoprotein A Repetitions Predominant
GCs	Germinal centers
GITR	Glucocorticoid-induced TNFR-related protein
GWAS	Genome-wide association studies
IC	Immune-complex
ICOS	Inducible costimulator
IFNs	Interferons
IgD	Immunoglobulin D
ILs	Interleukins
IRF4	Interferon Regulatory Factor 4
LAP	Latency-associated peptide
LDGs	Low-density granulocytes
MHC	Major histocompatibility complex
mTOR	Mammalian Target of Rapamycin
NETs	Neutrophil extracellular traps
NFAT	Nuclear factor of activated T-cells
NGS	Next generation sequencing
NK	Natural killer
NLE	Neonatal lupus erythematosus
PD-1	Programmed death-1
pDC	Plasmacytoid DC
PLC-γ2	Phospholipase C-γ2
PRRs	Pattern recognition receptors
ROCK	Rho-associated coiled-coil domain protein kinase
SFB	Segmented filamentous bacteria
SLAMF4	Signaling lymphocytic activation molecule family member 4
SLE	Systemic lupus erythematosus
SLEDAI	Systemic Lupus Erythematosus Disease Activity Index
TACI	Transmembrane activator, calcium modulator and cyclophilin ligand interactor
Tfh	T follicular helper
Tfr	Follicular regulatory T cells
TGF-β	Transforming growth factor-b
Th	T helper lymphocytes
Th1	T helper 1 cells
Th17	T helper 17 lymphocytes
TLRs	Toll-like receptors
TNF	Tumor necrosis factor
Tregs	Regulatory T cells
